# Real Assessment of Maximum Oxygen Uptake as a Verification After an Incremental Test Versus Without a Test

**DOI:** 10.3389/fphys.2021.739745

**Published:** 2021-10-28

**Authors:** Paulina Hebisz, Agnieszka Danuta Jastrzębska, Rafał Hebisz

**Affiliations:** Department of Physiology and Biochemistry, University School of Physical Education in Wrocław, Wrocław, Poland

**Keywords:** maximum oxygen uptake, VO_2_ plateau, physical fitness, cycle ergometer, verification phase, incremental test

## Abstract

The study was conducted to compare peak oxygen uptake (VO_2peak_) measured with the incremental graded test (GXT) (VO_2__peak_) and two tests to verify maximum oxygen uptake, performed 15 min after the incremental test (VO_2__peak__1_) and on a separate day (VO_2__peak__2_). The aim was to determine which of the verification tests is more accurate and, more generally, to validate the VO_2__max_ obtained in the incremental graded test on cycle ergometer. The study involved 23 participants with varying levels of physical activity. Analysis of variance showed no statistically significant differences for repeated measurements (*F* = 2.28, *p* = 0.118, η^2^ = 0.12). Bland–Altman analysis revealed a small bias of the VO_2__peak__1_ results compared to the VO_2__peak_ (0.4 ml⋅min^–1^⋅kg^–1^) and VO_2__peak__2_ results compared to the VO_2__peak_ (−0.76 ml⋅min^–1^⋅kg^–1^). In isolated cases, it was observed that VO_2__peak__1_ and VO_2__peak__2_ differed by more than 5% from VO_2__peak_. Considering the above, it can be stated that among young people, there are no statistically significant differences between the values of VO_2peak_ measured in the following tests. However, in individual cases, the need to verify the maximum oxygen uptake is stated, but performing a second verification test on a separate day has no additional benefit.

## Introduction

Maximum oxygen uptake (VO_2__max_) is considered to be the gold standard in assessing oxygen capacity, as it reflects the efficiency of the respiratory and circulatory system and the efficiency of the muscular system in using oxygen whilst exercising (Bassett and Howley, 2000; Lucia et al., 2001; Martino et al., 2002; Joyner and Coyle, 2008). The incremental graded test (GXT) protocol is commonly used to assess the VO_2__max_, which involves increasing the external load and continuing it until the subject reaches volitional exhaustion (Beltz et al., 2016). For years, the paradigm of the GXT was accepted and this form of VO_2__max_ testing was used. However, for several years, there has been a discussion of whether the GXT in each case allows for an accurate measurement of maximum oxygen uptake (Howley et al., 1995; Poole et al., 2008; Sánchez-Otero et al., 2014; Schaun, 2017). It was pointed out that subjects with no experience for maximal efforts and those with low motivation and low cardiorespiratory fitness may interrupt the test before reaching VO_2__max_ due to fatigue-related symptoms (Midgley et al., 2007b; Poole and Jones, 2017).

Therefore, new criteria for the accuracy of VO_2__max_ measurements have been proposed (Howley et al., 1995; Sánchez-Otero et al., 2014; Beltz et al., 2016; Schaun, 2017). It has been suggested that achieving a VO_2_ plateau in the final phase of the GXT is proof that a VO_2__max_ measurement is accurate (Howley et al., 1995). However, it has been documented that in many subjects (both athletes and non-athletes), it is impossible to separate the plateau phase when reaching VO_2__max_ (Lucia et al., 2006; Schaun, 2017; Hebisz et al., 2018). The other criteria for accurately measuring VO_2__max_–analysis of peak respiratory quotient, peak heart rate (HR), and post-workout lactate concentration–have also been widely discussed (Howley et al., 1995; Duncan et al., 1997; Beltz et al., 2016). Nonetheless, their high inter-subject variability may suggest that some subjects do not satisfy mentioned criterions even if their maximum effort is made, which lowers their value. It has been also demonstrated that the criterion of achieving a VO_2_ plateau in the final phase of the GXT frequently does not meet the criteria for HR and lactate concentration (Poole et al., 2008). These limitations reduce the certainty that subjects performing the GXT reach their “true” VO_2__max_.

Considering the doubts about the effectiveness of the above-mentioned criteria in verifying the accuracy of VO_2__max_ measurements, constant power verification tests were proposed (Midgley et al., 2006; Beltz et al., 2016; Poole and Jones, 2017; Schaun, 2017; Possamai et al., 2020). The idea is simply to provoke the VO_2_ plateau through constant-load effort performed with intensities ranging from submaximal to supramaximal effort (Barker et al., 2011; Nolan et al., 2014; Poole and Jones, 2017; Astorino and DeRevere, 2018). Usually, the verification tests are performed approximately 5–15 min after the incremental test (Schaun, 2017) and last several minutes (Barker et al., 2011; Nolan et al., 2014; Beltz et al., 2016; Schaun, 2017; Astorino and DeRevere, 2018).

On the other hand, Possamai et al. (2020) suggests that the test to verify the VO_2__max_ obtained in the GXT should be performed on a different day, assuming that the subject’s exercise tolerance/capacity is higher then and that the peak oxygen uptake (VO_2__peak_) measured in a verification test on another day are not lower than that from a verification test performed several minutes after the GXT. However, in both verification tests they used a power output level of 100% of maximal power–as measured in a previous incremental test–which could have contributed to similar values of oxygen uptake being recorded in the tests. Moreover, their results showed that the VO_2__peak_ achieved in the verification test performed on a separate day were closer to the VO_2__peak_ of the GXT than that of a verification test done several minutes after the GXT.

More recently, in order to verify the VO_2__peak_ from the GXT, researchers proposed performing the verification test with a power level exceeding the power output of the GXT, but mainly several minutes after the GXT (Barker et al., 2011; Nolan et al., 2014; Schaun, 2017; Astorino and DeRevere, 2018). It seems that it would be worth using a higher load in the verification test performed on a separate day, as exercise tolerance is higher then.

The aim of this study was to compare the values of VO_2__peak_ obtained from the incremental test and from two verification tests completed with a power output of 110% of the peak power output reached in a previous incremental test [the first one was performed 15 min after the progressive test (T*_ver–__1_*), whilst the second one was performed on a separate day (T*_ver–__2_*)]. It was hypothesized that in individual cases, the verification test performed on a separate day may allow for higher VO_2__peak_ values than the incremental test and the verification test performed several minutes after the incremental test.

## Materials and Methods

The study involved 23 participants: recreationally active individuals (*n* = 13, including 7 women and 6 men) and athletes (cyclists) (*n* = 10, including 4 women and 6 men). Each participant had been active recreationally or practicing sport (cyclists) for at least 3 years. The two groups, the recreationally active people and the athletes, were similar in regard to their anthropometric characteristics, whereas the parameters for physical capacity–VO_2__peak_ (*p* < 0.000) and power value (P_max_) (*p* < 0.000) differed significantly (Table 1).

**TABLE 1:** Basic anthropological and physiological parameters characterizing the subjects.

	**All (*n* = 23)**	**Recreational active (*n* = 13)**	**Athletes (*n* = 10)**	**Females (*n* = 11)**	**Males (*n* = 12)**
Age (years)	22.003.79	21.231.01	23.005.64	21.643.67	22.334.03
Body height (m)	1.740.10	1.760.11	1.720.08	1.670.06	1.820.07^*^
Body mass (kg)	68.509.96	70.6411.38	65.737.39	61.756.92	74.698.22^*^
VO_2peak1_ (ml⋅kg^–1^⋅min^–1^)	52.0013.31	42.626.10	64.189.58^*^	45.468.44	57.9814.42^*^
Pmax (W)	288.9177.71	244.2356.26	347.0062.51^*^	230.6449.31	342.3357.94^*^

*VO_2__*peak1*_, the peak oxygen uptake in an incremental test; Pmax, the maximum aerobic power measured during the progressive test; data are presented as mean ± standard deviation.*

*p < 0.05 for the difference between groups.

The study design was approved by the institutional review board and was conducted in accordance with the ethical standards established by the Declaration of Helsinki. Written informed consent was obtained from all participants after the study details, procedures, benefits, and risks were explained.

### Exercise Tests

**FIGURE 1:**
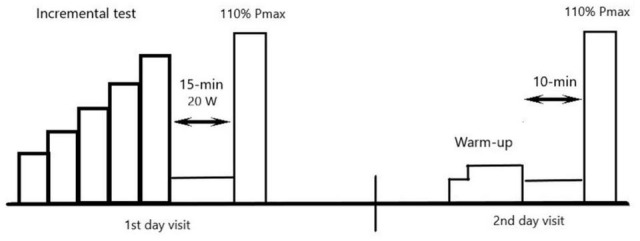
Scheme of visit in laboratory.

The study consisted of three exercise tests (Figure 1). On the first day of the study, each participant performed an incremental graded test (GXT) and a verification test (T*_ver–__1_*). After a 48-h break, an additional verification test (T*_ver–__2_*) was performed, which was only preceded by a warm-up. The tests (GXT and T*_ver–__1_*) and T*_ver–__2_* were performed at a similar time of day (±30 min). All the tests were carried out using a Lode Excalibur Sport electronically braked cycloergometer (Lode BV, Groningen, Netherlands). The tests were performed in controlled laboratory conditions at an exercise laboratory (PN-EN ISO 9001:2001 certified). One week prior to the incremental graded test, the participants were familiarized with the protocol of the test.

### Incremental Exercise Test With Verification Test Performed on the Same Day

The VO_2__peak_ was determined using a continuous GXT, with a self-selected pedal rate no lower than 60 rev/min. The test started with a 40-W or 50-W load (for women and men, respectively), and it was increased by 40 W or 50 W (for women and men, respectively) every 3 min until volitional exhaustion. Heart rate was recorded with a V800 cardiofrequencimeter (Polar, Oy, Finland). The respiratory parameters were measured breath-by-breath (Quark, COSMED, Milan, Italy) and averaged over 30-s intervals. The data recording began 2 min before GXT and ended 5 min after the verification test (T*_ver–__1_*). The device was calibrated with an atmospheric air and gas mixture: 5% CO_2_, 16% O_2_, and 79% N_2_. Oxygen uptake (VO_2_), exhaled carbon dioxide (VCO_2_), and minute pulmonary ventilation (VE) were measured. The highest VO_2_ recorded in the GXT was taken as the VO_2__peak_, whilst the highest VO_2_ recorded in the T*_ver–__1_* was taken as the VO_2__peak__1_.

Based on the respiratory data records from the GXT, the first ventilatory threshold (VT1) was determined at the point preceding the first non-linear increase in VE⋅VO_2_^–1^ without a concomitant increase in VE⋅VCO_2_^–1^ equivalent; the second ventilatory threshold (VT2) was at the point preceding the second non-linear increase in VE⋅VO_2_^–1^ accompanied by an increase of VE⋅VCO_2_^–1^ equivalent, according to the methodology described by Davis et al. (1980) and Beaver et al. (1986).

The cycloergometer was controlled by a computer, which recorded instantaneous power and exercise time. The maximum aerobic P_max_ was obtained by subtracting 0.22 W for women and 0.28 W for men for each missing second of the last performed load. After the end of the test, the subject rested for 15 min, with an active rest on a 20-W cycloergometer. Next, a 3-min, square-wave T*_ver–__1_* was performed with an intensity of 110% of P_max_ with regards to Schaun (2017).

### Verification Test Performed on a Different Day

The test was preceded by a 15-min warm-up consisting of 5 min of exercise at an intensity corresponding to the power achieved with the VT1, then 10 min at a power corresponding to half the distance between the VT1 and the VT2. The warm-up was followed by a 10-min passive break. T*_ver–__2_* was 3 min long and was performed at an intensity of 110% of P_max_, as determined by the results of the incremental graded test performed 2 days prior. The recording of respiratory parameters started 1 min before the verification test and ended 5 min after it was completed. The values averaged every 30 s were used in data analysis. The highest recorded oxygen uptake (from the averaging of 30-s intervals) was taken as the VO_2peak_ in the verification test performed on a separate day (VO_2__peak__2_).

### Statistical Analysis

The differences (expressed in %) between VO_2__peak_ and VO_2__peak__1_, as well as between VO_2__peak_ were calculated for each participant. The tolerance of measurement error was at 5% (Midgley et al., 2007a; Romero-Fallas et al., 2012; Hall-Lopez et al., 2015). Data normality was assessed through the Kolmogorov–Smirnov test with Lilliefors significance correction. Bland–Altman analysis was performed to determine the size of the difference shift between VO_2__peak_ and VO_2__peak__1_, as well as between VO_2__peak_ and VO_2__peak__2_. Pearson’s correlation and linear regression were performed for comparing the results of GXT and T*_ver–__1_* or T*_ver–__2_*. STATISTICA 13.1 software (StatSoft Inc., Tulsa, OK, United States) was used for further statistical processing of the data. All data are reported as mean ± SD. Analysis of variance with repeated measurements and the Scheffe *post hoc* test were used to determine whether factors such as sex, athletic ability, or subsequent tests affected VO_2peak_. The results were considered statistically significant at an alpha level of *p* < 0.05.

## Results

The GXT and T*_ver–__2_* were performed by 23 participants, while T*_ver–__1_* was performed by 21 participants (2 participants refused to perform this test because of perceived fatigue).

The analysis of the main effects showed statistically significant differences in oxygen uptake for sex (*F* = 25.02; *p* = 0.000; η^2^ = 0.60) and physical activity level (*F* = 74.24; *p* = 0.000; η^2^ = 0.81). There were no statistically significant differences for repeated measurements (*F* = 2.28, *p* = 0.118, η^2^ = 0.12) or mixed effects for repeated measurements and sex (*F* = 0.68, *p* = 0.516, η^2^ = 0.04), nor for mixed effects for repeated measurements and physical activity level (*F* = 0.20, *p* = 0.820, η^2^ = 0.01) (Table 2).

**TABLE 2:** Peak oxygen uptake value in the incremental test and in the verification tests in the entire group of subjects, as well as after dividing the group according to sex and physical activity level.

	**Peak oxygen uptake (VO_2_peak) [ml⋅min^–1^⋅kg^–1^]**		
	**Progressive test (*n* = 23)**	**Verification test 1 (*n* = 21)**	**Verification test 2 (*n* = 23)**
Whole group (*n* = 23^)	51.99 ± 13.31	51.03 ± 13.73	52.75 ± 13.37
Females (*n* = 11^)	45.46 ± 8.44	44.09 ± 7.79	45.08 ± 7.67
Males (*n* = 12^)	57.98 ± 14.42	57.35 ± 15.19	59.78 ± 13.85
Athletes (*n* = 10^)	64.18 ± 9.58	64.94 ± 10.30	64.52 ± 10.58
Recreationally active (*n* = 13)	42.62 ± 6.10	42.48 ± 6.66	43.70 ± 6.32

*Data are presented as mean ± standard deviation.*

*^∧^-21 participants completed the verification test 1, two athletes (one woman and one man) refused to participate in this test.*

**FIGURE 2:**
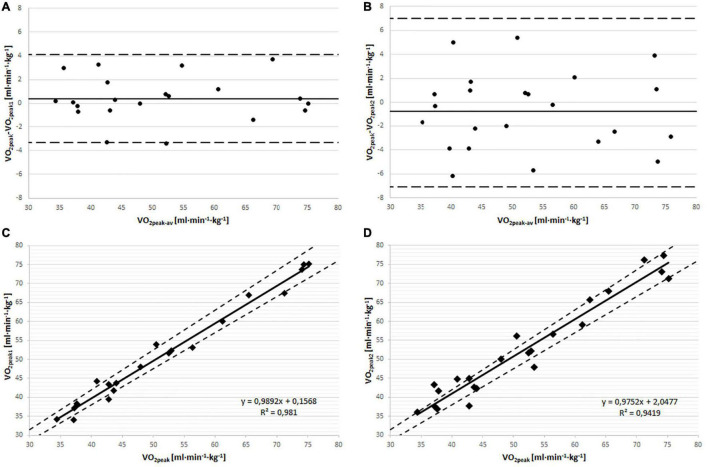
Bland-Altman plot showing: **(A)** Individual differences between the VO_2peak_ values attained in the incremental and VO_2peak1_ from T*_ver–1_* **(B)** individual differences between the VO_2peak_ values attained in the incremental and VO_2peak2_ from T*_ver–2_*. Solid line show bias and dashed lines represent a 1.96 SD (standard deviation) for difference between peak oxygen uptakes. **(C)** Pearson correlation between VO_2peak_ and VO_2peak1_. **(D)** Pearson correlation between VO_2peak_ and VO_2peak2_. In **(C,D)** the dashed lines indicate the 5% threshold difference from VO_2peak_.

The individual analysis showed that 2 subjects in the T*_ver–__1_* and 7 subjects in the T*_ver–__2_* had a higher VO_2peak_ by 5% than in the GXT (Table 3). Bland–Altman analysis (Figure 2) revealed a small bias of the VO_2__peak__1_ results compared to the VO_2__peak_ (0.4 ml⋅min^–1^⋅kg^–1^) and VO_2__peak__2_ results compared to the VO_2__peak_ (−0.76 ml⋅min^–1^⋅kg^–1^).

**TABLE 3:** The number of people who achieved a lower, higher or equal peak oxygen uptake in the verification tests compared to the peak oxygen uptake achieved in the progressive test.

	**Whole**	**I division**		**II division**	
	**Group (*n* = 23)**	**Females (*n* = 11)**	**Males (*n* = 12)**	**Athletes (*n* = 10)**	**Recreationally active (*n* = 13)**
VO_2__peak_ < VO_2__peak__1_	2	1	1	0	2
VO_2__peak_ > VO_2__peak__1_	4	2	2	2	2
VO_2__peak_ = VO_2__peak__1_	15	7	8	6	9
VO_2__peak_ < VO_2__peak__2_	7	2	5	2	5
VO_2__peak_ > VO_2__peak__2_	3	2	1	2	1
VO_2__peak_ = VO_2__peak__2_	13	7	6	6	7

*The analysis was performed taking into account the division of the study group according to sex (I) and physical activity level (II).*

*VO_2__peak_, the peak oxygen uptake in the progressive test; VO_2__peak__1_, the peak oxygen uptake in the verification test 1; VO_2__peak__2_, the peak oxygen uptake in the verification test 2; <, less than…; >, greater than…; =, equal….*

The raw test records that were performed in the studies described in this work are posted in the repository at https://repod.icm.edu.pl/dataset.xhtml?persistentId=doi:10.18150/HGE2PK.

## Discussion

In order to assess the VO_2__peak_, researchers traditionally use the GXT test until exhaustion. Since the primary criterion of VO_2__peak_ attainment–a VO_2_ plateau in exhaustion–is not always reached during the GXT, some researchers have postulated using subsequent verification tests (Niemelä et al., 1980; Midgley et al., 2007b; Poole and Jones, 2017). However, in the available literature, there are contradictory suggestions as to the need for verification tests. There are opinions that question the validity of performing tests to verify the VO_2__max_ obtained from a progressive test, due to the minimal individual differences between the results of progressive and verifying tests (Rossiter et al., 2006; Murias et al., 2018; Brito et al., 2019). Similar results, confirmed by Bland–Altman analysis, were presented by McGawley (2017) when he compared the VO_2__peak_ measured in the progressive test with the VO_2__peak_ measured in a 4-min time trial run, performed on a separate day. The data presented herein show no differences in mean VO_2__peak_ in the GXT and T*_ver–__1_* versus T*_ver–__2_* (Table 2). Bland–Altman analysis showed a small bias of VO_2__peak__1_ compared to VO_2__peak_, as well as of VO_2__peak__2_ compared to VO_2__peak_ (Figure 2). However, several subjects (both recreationally active people and athletes) achieved higher VO_2__peak__1_ or VO_2__peak__2_ values than VO_2__peak_. Therefore, we support the postulate of Poole and Jones (2017) about the need to perform tests verifying the values of VO_2peak_ measured in progressive tests.

In most available literature, VO_2__max_ verifier tests are performed on the same day as the progressive test (Midgley et al., 2007b; Astorino, 2009; Kirkeberg et al., 2011; Dalleck et al., 2012; Poole and Jones, 2017; Adam et al., 2018). The factor differentiating used procedures is the time between the tests. Intervals of between 5 and 15 min have commonly been used (Midgley et al., 2007b; Poole and Jones, 2017; Adam et al., 2018), although intervals ranging from 1 to 3 min (Kirkeberg et al., 2011) to even 60–90 min (Astorino, 2009; Dalleck et al., 2012; Nolan et al., 2014) have been used for verification tests performed on the same day. Nolan et al. (2014) reported no differences in VO_2__peak_ between verification tests performed with 105% P_max_ after 20- and 60-min recovery periods. Thus, 20 min of recovery may be sufficient for physically active subjects. As noted by Scharhag-Rosenberger et al. (2011), comparable VO_2__peak_ values after an incremental test and verification test followed by a 10-min break indicates that even shorter breaks can be used. The results reported by Kirkeberg et al. (2011) show that even short recovery periods of 1–3 min turned out to be sufficient among physically active people. Regardless of the intervals used between the tests, it seems that the effectiveness of the VO_2__max_ verification tests we quote above was similar. Therefore, it could be concluded that VO_2__peak_ in a verification test seems not to be affected by the exhaustion caused by the incremental test. Schaun (2017) also stated that the time elapsed between an incremental test and a verification test is not a key aspect to achieving the verification criterion. Attempts were also made to perform tests to verify VO2max on a different day than the progressive test (Scharhag-Rosenberger et al., 2011; Possamai et al., 2020; Sawyer et al., 2020). Possamai et al. (2020) found that during the verification test performed on a separate day, the exercise capacity is greater than during the verification test performed several minutes after the progressive test. Such a conclusion was formulated on the basis of a longer effort time in a verification test performed on a separate day, compared to a test performed several minutes after the progressive test. However, the greater exercise capacity described by Possamai et al. (2020) did not affect the VO_2__peak_ values, which were similar in individual tests. Scharhag-Rosenberger et al. (2011) also performed verification tests on a separate day. Based on the results of these studies, it was also considered that VO_2__peak_ in the verification test performed on a separate day does not differ significantly from VO_2__peak_ from the verification test performed several minutes after the progressive test. However, in the studies described above, verification tests were preceded by a short warm-up.

Another factor that may influence VO_2__peak_ values is the type of warm-up used before the verification test carried out on a separate day. Possamai et al. (2020) preceded the verification test with a warm-up of 6 min and measured the power at the lactate threshold, defined as the first sharp increase in lactate concentration in a progressive test. An even shorter warm-up, lasting 5 min, was used by Scharhag-Rosenberger et al. (2011) and Sawyer et al. (2020). In Scharhag-Rosenberger et al. (2011) study the warm-up was done at a speed higher than the lactate threshold speed. Also, a warm-up in the research of Sawyer et al. (2020) consisted of 5 min of exercise, however, at an intensity of 50 W (men) or 30 W (women) which is lower than those proposed by Scharhag-Rosenberger et al. (2011). Bishop (2003) stated that the optimal warm-up duration before intensive efforts with an average duration should be at least 10 min, which allows the subject to reach steady-state VO_2_. In our own studies, the warm-up lasted 15 min, including 5 min of VT1 effort and 10 min of effort measured halfway between VT1 and VT2. We concluded that such a warm-up, performed before the verification test on a separate day, may allow to obtain higher VO_2__peak_ values than in the above-cited works (Scharhag-Rosenberger et al., 2011; Possamai et al., 2020; Sawyer et al., 2020). This assumption was supported by the results of our own previous studies (Hebisz et al., 2017), in which we also used a long warm-up time. We then found that it is possible to achieve a higher VO_2__peak_ value even during a series of four short sprints (30-s each) in comparison to the progressive test. However, analysis of variance showed no statistically significant differences between VO_2__peak_, VO_2__peak__1_ and VO_2__peak__2_ in the entire group of subjects. Moreover, Bland-Altman analysis revealed a bias of VO_2__peak__1_ compared to VO_2__peak_, as well as of VO_2__peak__2_ compared to VO_2__peak_ was neglectable. Therefore, the research procedure we used produced similar statistical effects as the research results described by Scharhag-Rosenberger et al. (2011).

The possibility that the training level meets the VO_2__peak_ verification criterion was also analyzed in this study. The above-cited studies (Scharhag-Rosenberger et al., 2011; Nolan et al., 2014; Possamai et al., 2020) involved physically active people, but they were not professional athletes. Only in a review, Costa et al. (2021) stated that concordance between VO_2__peak_ level from GXT and verification tests is not affected by the cardiorespiratory level of participants. In the present study, we compared athletes with recreationally active subjects. Analysis of variance showed no mixed effects on repeated measurements and level of physical activity. Therefore, the results of the studies described in this work support Costa et al.’s (2021) suggestion that the effects of VO_2__max_ verification are not related to the level of efficiency (cardio-respiratory level).

## Limitations

In our research, we compared VO_2__peak_ values achieved by cyclists and amateurs. In this way, our research complements the knowledge about the effects of verification tests, because so far there has been little information in the literature about the results of verification tests performed by athletes. On the other hand, performing analyses on a group of respondents consisting of cyclists and amateurs is a factor limiting the certainty of our conclusions, because athletes and amateurs are characterized by a different level of physical performance (muscular power, VO_2__*peak*,_ VO_2__max_). Different levels of exercise tolerance in our studies may affect the high variability of the obtained results and thus may affect the results of statistical analyses.

The second factor limiting the certainty of our conclusions is the way the subjects are prepared for the verification test performed on a separate day. After warming up, and before the verification test, we used a passive break of 15 min. We decided that this way of preparing for the test is good, because in the literature there are suggestions that the type of break (active or passive) before a few minutes and intense efforts does not affect exercise capacity (McAinch et al., 2004; Fennell and Hopker, 2021). In addition, vasodilation of muscle vessels and the activity of histamine H1 and H2 receptors is high even for 90 min after exercise (Luttrell and Halliwill, 2017). However, the use of a passive break before the verification test performed on a separate day may have resulted in high variability of VO_2__peak_–VO_2__peak__2_.

## Conclusion

Among young people, there were no statistically significant differences between VO_2peak_ measured in the progressive test and VO_2peak_ measured in the verification tests (performed 15 min after the progressive test and performed on a separate day), in general. There are also no differences in peak oxygen consumption between the progressive test and the verification tests after dividing the group into athletes and recreationally active individuals in any of the above-mentioned groups. In individual cases, the need to verify the maximum oxygen uptake is stated, but performing a second verification test on a separate day does not bring additional benefits.

## Data Availability Statement

The datasets presented in this study can be found in online repositories. The names of the repository/repositories and accession number(s) can be found below: https://repod.icm.edu.pl/dataset.xhtml?persistentId=doi:10.18150/HGE2PK.

## Ethics Statement

The studies involving human participants were reviewed and approved by the Senate Research Ethics Committee at University School of Physical Education in Wrocław. The patients/participants provided their written informed consent to participate in this study.

## Author Contributions

PH contributed to the study design and data collection, and drafted the manuscript. AJ contributed to the data collection and made the critical revisions to the manuscript. RH contributed to the study design and data analysis, and drafted the manuscript. All authors discussed the results, commented and edited the manuscript at all stages, approved the final version and agreed to be accountable for all aspects of the work.

## Conflict of Interest

The authors declare that the research was conducted in the absence of any commercial or financial relationships that could be construed as a potential conflict of interest.

## Publisher’s Note

All claims expressed in this article are solely those of the authors and do not necessarily represent those of their affiliated organizations, or those of the publisher, the editors and the reviewers. Any product that may be evaluated in this article, or claim that may be made by its manufacturer, is not guaranteed or endorsed by the publisher.
